# Quinine with Nervous Sedatives

**Published:** 1881-02

**Authors:** 


### QUININE WITH NERVOUS SEDATIVES.

AS A result of extended chemical experience in the *use of quinine with nervous sedatives*, the author believes, that quinine will increase the sedative effect of the bromides, belladonna, and hyoscyamia, while it simultaneously decreases or dispels the depression which these medicines usually produce; he does not attempt any explanation of the *modus operandi.* In epileptics, it is his habit to give the bromides until bromism has been induced, and then to give, together with them, two or three grains of quinine three times daily. He admits that there are some epileptics, of excellent general health, to whom quinine seems injurious, and who bear full sedation with the bromides very well; but he insists that these are very exceptional cases. He adds quinine to considerable doses of the sedatives above mentioned, in all cases where the patients are pale, languid, and anæmic.

